# Effect of Crosslinking Temperature on the Insulation Performance of XLPE Secondary Crosslinking Insulation Interface Layer

**DOI:** 10.3390/polym17070936

**Published:** 2025-03-30

**Authors:** Ming Hu, Hongliang Zhang, Xufei Ge, Xiping Hu, Zehao Zhang, Xiaoyan Cao, Zerui Li, Wenbo Huo

**Affiliations:** 1 College of Mechanical and Electrical Engineering, Guangdong University of Technology, Guangzhou 510006, China; 2 Zhongtian Technology Submarine Cable Co., Ltd., Nantong 226000, Chinagexf@ztt.cn (X.G.); marco.hu@zttgroup.com (X.H.);; 3 College of Electrical Engineering, Sichuan University, Chengdu 610065, China

**Keywords:** crosslinking temperature, cross-linked polyethylene, insulation interface layer, insulation performance, factory connector

## Abstract

To investigate the impact of temperature variations during the secondary crosslinking of cross-linked polyethylene (XLPE) on the insulation performance of the interface layer, commercial XLPE cable pellets were subjected to flat hot pressing at 140 °C, 160 °C, 180 °C, and 200 °C. XLPE insulation interface layers were prepared at different secondary crosslinking temperatures. The samples were characterized by gel content, differential scanning calorimetry (DSC), thermal elongation test, power frequency breakdown voltage, and scanning electron microscope (SEM). Key parameters, including crosslinking degree, crystallinity, thermal elongation, and characteristic breakdown voltage of the samples, were obtained. The results indicate that as the secondary crosslinking temperature increases, the crosslinking degree of the samples rises, while thermal elongation initially decreases and then increases. Crystallinity shows a decreasing trend overall. The characteristic breakdown voltage shows a trend of increasing first and then decreasing. When the temperature is 180 °C, the characteristic breakdown voltage reaches its maximum value. Therefore, increasing the secondary crosslinking temperature can help improve the mechanical and electrical properties of the XLPE insulation interface layer. However, crystallinity decreases at excessively high temperatures, which has a negative impact on insulation performance.

## 1. Introduction

Submarine cables are critical infrastructure for global communication, data, and energy transmission, as well as essential components for cross-sea power transmission [1,2,3]. With the ongoing implementation of national marine development strategies, the construction of power grid interconnection projects between coastal islands and the mainland, offshore wind power farms, nuclear power plants, and drilling platforms has generated significant demand for submarine cables [4,5]. This trend reflects the continuous growth in submarine cable requirements [6]. Cross-linked polyethylene (XLPE) submarine cables are widely used for power transmission in fields such as wind power grid connection, island power supply, and long-distance cross-sea transmission.

The distance between offshore infrastructure and the mainland typically ranges from hundreds of kilometers to even greater distances [7]. However, due to limitations in manufacturing technology, the length of a single submarine cable is generally limited to about ten kilometers. To meet the length requirements, joint technology is employed to connect XLPE submarine cables, which has become an essential component of submarine cable transmission systems. Factory joints are commonly used in these systems [8,9]. The reliability of their quality is crucial for the safe and efficient operation of the entire submarine cable network. The use of secondary injection molding technology in the production of factory joints results in an insulation interface layer between the insulation recovery and the main insulation body [10,11]. This interface can easily lead to the accumulation of space charges [12,13], accelerating material degradation and potentially inducing insulation breakdown [14,15]. Therefore, studying the XLPE insulation interface layer in submarine cable factory joints and understanding the relationship between key production process parameters and performance changes is of great significance for the advancement of long-distance and high-capacity submarine cables.

The submarine cable factory joint is primarily formed by injecting newly melted XLPE into the insulation of the submarine cable body, which has been shaped into a cone in a factory or equivalent environment. It then undergoes secondary vulcanization under high temperature and pressure, ultimately forming the restored insulation of the factory joint [16]. During this vulcanization process, dicumyl peroxide (DCP) decomposes thermally to crosslink polyethylene as the crosslinking agent [17]. Therefore, the secondary vulcanization process is the key to determining the performance of the recovered insulation zone in submarine cable factory joints. The insulation performance of these joints is primarily influenced by crosslinking process parameters such as crosslinking temperature and pressure. Domestic cable manufacturers typically determine the crosslinking temperature and pressure based on production experience and type testing, with a typical crosslinking temperature of approximately 180 °C and a crosslinking pressure of around 1 MPa. At present, both domestic and international researchers predominantly focus on the effects of sulfurization pressure, degassing time [18,19,20,21], and interface roughness [22] on the conductivity, DC breakdown, and other properties of the insulation interface layer in factory joints. However, the crosslinking temperature during the secondary crosslinking process of factory joints directly affects the decomposition rate of crosslinking agents, resulting in significant variations in the crosslinking degree of the recovery insulation zone. These variations result in observable differences in the insulation properties of the factory joints [23]. The influence of crosslinking temperature on the physicochemical and electrical properties of the insulation material in the recovery insulation zone [24,25], as well as the underlying mechanisms, remains unclear [26]. Investigating the effects of crosslinking temperature and elucidating its role in the performance of the factory joint recovery insulation zone is of great significance for the development and engineering application of long-distance XLPE submarine cable systems operating at voltages of 500 kV and above.

This study produced insulation interface samples of submarine cable factory joints under crosslinking temperatures of 140 °C, 160 °C, 180 °C, and 200 °C. Through crosslinking degree testing, thermal extension experiments, breakdown field strength testing, and scanning electron microscopy observation, the influence of crosslinking temperature on the interface insulation performance of the factory joint was investigated. The results provide a theoretical basis for the development and production of submarine cable factory joints.

## 2. Materials and Methods

### 2.1. Materials and Sample Preparation

**Figure 1:**
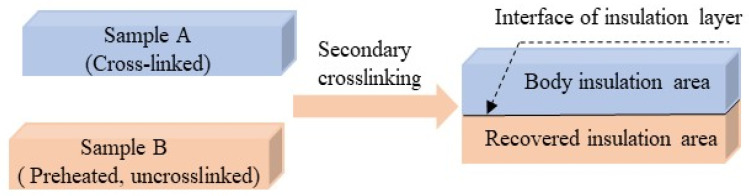
Preparation schematic diagram of insulation interface samples at different crosslinking temperatures.

The recovered insulation production process of factory joints was simulated in the laboratory using a flat hot pressing method to press commercial XLPE cable particles. The XLPE cable particles are composed of low-density polyethylene (LDPE), dicumyl peroxide, and auxiliary additive (including antioxidants). Factory joint insulation interface layer samples were prepared at different crosslinking temperatures. A schematic diagram of the insulation interface layer samples at different crosslinking temperatures is shown in Figure 1. The specific process is as follows: (1) A certain amount of XLPE cable particles are placed in a 50 mm × 50 mm × 10 mm mold and preheated at 120 °C under 4.5 MPa pressure for 30 min using a flat vulcanizing machine. The materials are then crosslinked at 180 °C for 30 min under 10 MPa pressure. When cooling to room temperature, sample A of the simulated cable body is obtained, with dimensions of 50 mm × 50 mm × 10 mm; (2) A small amount of XLPE particles are placed in the mold and preheated at 120 °C and 4.5 MPa pressure for 30 min on a flat vulcanizing machine. Sample A from step (1) is then pressed onto the preheated XLPE pellets and crosslinked respectively at 140 °C, 160 °C, 180 °C, and 200 °C under 1 MPa pressure for 30 min to obtain factory joint insulation interface samples, each with dimensions of 50 mm × 50 mm × 20 mm. (3) The prepared factory joint insulation interface samples are placed in a vacuum drying oven at 70 °C for 48 h to minimize the influence of crosslinking by-products as much as possible.

### 2.2. Calculation of the Crosslinking Degree

**Figure 2:**
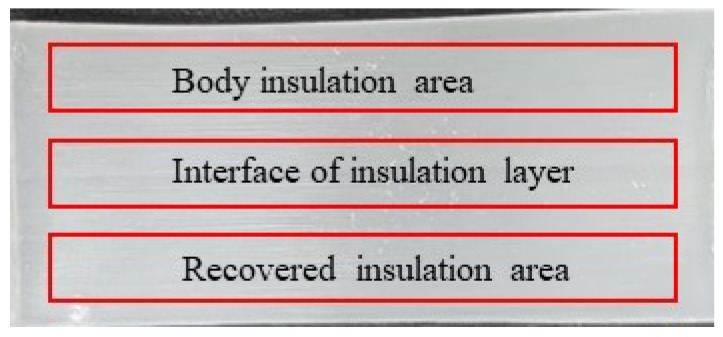
Sampling location annotation.

According to JB/T 10437-2004 [27], the crosslinking degree of XLPE is characterized by the gel content extraction method. The prepared sample was handled as shown in Figure 2 during the experiment. A sample with an initial mass of *M* was placed into a mesh bag with a 120-mesh metal sieve, denoted as *M*_2i_. The mesh bag containing the sample was soaked in xylene solvent and heated to 120 °C in an oil bath for 10 h. After heating, the mesh bag was placed in a 60 °C oven and dried for an additional 10 h. The metal mesh bag containing the sample was then weighed, and the mass was recorded as *M*_3i_. The principle of the gel content method is based on the extraction of a specific mass of XLPE by heating for a designated time, followed by calculating the percentage of residue before and after extraction to determine the crosslinking degree of the XLPE sample. The specific calculation is given in Formula (1):

(1) $$G=\frac{M_{3i}-M_{2i}+M}{M}\times 100\%$$

In the formula, *G* represents the crosslinking degree of the sample. *M* is the original weight of the sample (approximately 0.5 g). *M*_2i_ is the total mass of the sieve containing the sample before the reaction, and *M*_3i_ is the total mass of the sieve containing the sample after the reaction. Five tests were performed on the same group of samples, then the maximum and minimum values were discarded. The average of the remaining values was taken as the measured result of crosslinking degree.

### 2.3. Crystallinity Test

Differential scanning calorimetry (DSC) was performed using a DSC3 platform of Mettler Toledo in Changzhou, China to characterize the crystallization and melting properties. The sample processing and heating procedure contains four steps: (1) The insulation interface samples prepared at different crosslinking temperatures were cut into small pieces of approximately 5 mg and placed in a crucible for testing; (2) The sample was placed in a nitrogen environment; (3) The temperature range during testing was 25–140 °C, with a heating rate of 10 °C/min. (4) The temperature was maintained at 140 °C for three minutes, during which the variations in heat flow were monitored. To minimize random interference, each group of samples was tested than three times. Based on the obtained heat flux temperature curve, the crystallinity of the insulation interface layer samples used Formula (2) to calculate:

(2) $$X_{c}=\frac{\Delta H_{m}}{\Delta H_{100}}\times 100\%$$

In the formula, *X*c represents the crystallinity. Δ*H*_m_ is the heat of melting, and Δ*H*_100_ is the enthalpy of melting when the crystallinity of the sample is 100%, which is taken as 287.3 J/g in this study.

### 2.4. Thermal Extension Experiment

**Figure 3:**
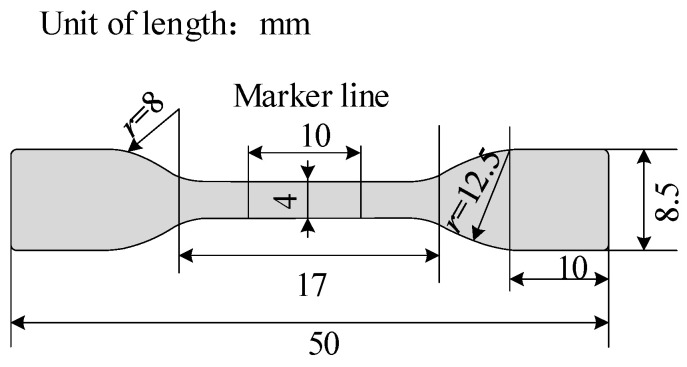
Dumbbell-shaped XLPE specimen.

The thermal extension test is an important indicator for testing the mechanical and physical properties of cable insulation layers [28,29]. It can also serve as a reference indicator for assessing the crosslinking degree of XLPE samples. According to the GB/T 2951.11-2008 standard [30], the XLPE sample needs to be made into a dumbbell shape with a thickness of 1 mm before the experiment, and its specific size requirements are shown in Figure 3.

The main steps of the thermal extension experiment were as followed:

1. A vernier caliper was used to measure the cross-sectional area of the prepared dumbbell-shaped samples. The corresponding load capacity was then calculated for the given size.
2. The dumbbell-shaped samples were fixed onto the test bracket of the thermal extension experimental device, with a fixed mass applied below. Then the thermal extension experimental device was placed into an oven with temperature and humidity control (model: RGDJ-800, Yekeyin Experimental Equipment Co., Ltd., Chengdu, China), preheated to 200 °C. Timing began when the temperature reached 200 °C (approximately 10 min), accumulating for 15 min.
3. The distance between the marked lines of the dumbbell-shaped samples was recorded after 15 min. The thermal elongation was calculated using the following Formula (3):

(3) $$Thermal\;elongation(\%)=\frac{L-L_{0}}{L_{0}}\times 100\%$$

In the formula, *L*_0_ represents the initial distance between the marked lines in the middle of the dumbbell-shaped sample, with a fixed value of 10 mm. *L* represents the distance between the marked lines after 15 min of heating. Five tests were performed on the same group of samples in this experiment. Then the maximum and minimum values were discarded, and the average of the remaining data was taken as the result of the thermal extension experiment.

### 2.5. Breakdown Voltage Test

**Figure 4:**
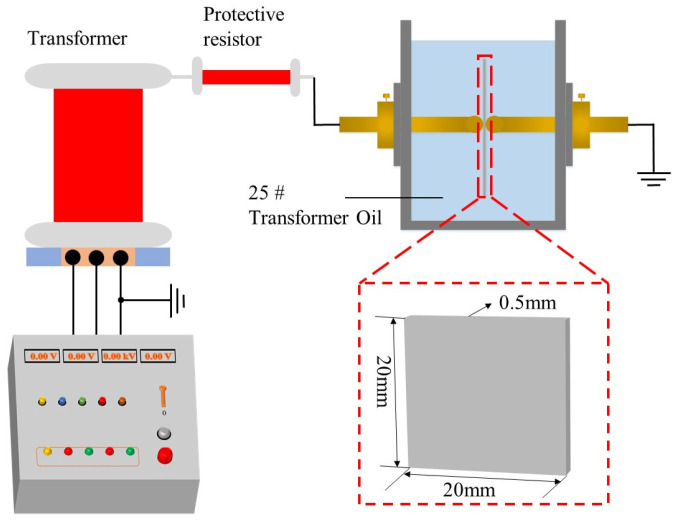
Power frequency breakdown voltage testing system.

A ball-ball electrode setup was employed for testing in the experiment. A thin sample containing an interface was placed between the two electrodes. Due to the small sample size, the sample was immersed in 25 # transformer oil to prevent surface discharge, as shown in Figure 4. The uniform voltage boosting method was adopted. Initially, the voltage was increased to 5 kV at a boosting rate of 500 V/s, followed by a ramp-up rate of 1 kV/min. The breakdown voltage was recorded when the sample experienced breakdown. Five samples were prepared for each group to perform the breakdown voltage tests.

Due to the inherent variability and randomness in breakdown voltage values, the Weber distribution was used for statistical analysis, in accordance with the recommendations of the International Electrotechnical Commission (IEC) 930 and the Chinese national standard GB/T 29310-2012 [31,32]. The two-parameter Weber distribution is shown in Formula (4). The voltage corresponding to a 63.2% breakdown probability is taken as the characteristic breakdown voltage.

(4) $$F\left(U,\;\alpha ,\;\beta \right)=1-\exp\left\{-{\left(\frac{U}{a}\right)}^{\beta }\right\}$$

where *U* is the breakdown voltage. *α* is the scale parameter, and *β* is the shape parameter.

### 2.6. Scanning Electron Microscopy Observation

To further investigate the microstructure of XLPE/XLPE interface at different crosslinking temperatures, scanning electron microscopy (SEM) was employed. Since XLPE is a high-molecular-weight polymer material, slicing knife marks at room temperature can affect the SEM morphology at the microscopic level. Therefore, the cross-sections were obtained by low-temperature quenching. The sample preparation procedure was as follows: (1) A strip-shaped sample was cut from the insulation interface, and a wedge-shaped notch was made at the tangential interface. (2) The strip sample was immersed in liquid nitrogen and frozen at low temperature for 10 min. (3) The frozen sample was quenched along the notch using a fixture. (4) To enhance the conductivity of the XLPE material for SEM analysis, a thin and uniform layer of gold solution was sputtered onto the sample surface using an ion sputtering instrument.

The JSM-7500F scanning electron microscope, produced by Japan Electronics Corporation in Tokyo, Japan, was used for observation. The resolution of this device can be adjusted between 1.0 nm (at 15 kV) to 1.4 nm (at 1 kV), with a magnification range of 25 to 100,000 times.

## 3. Results

### 3.1. Analysis of Crosslinking Degree Test Results

**Figure 5:**
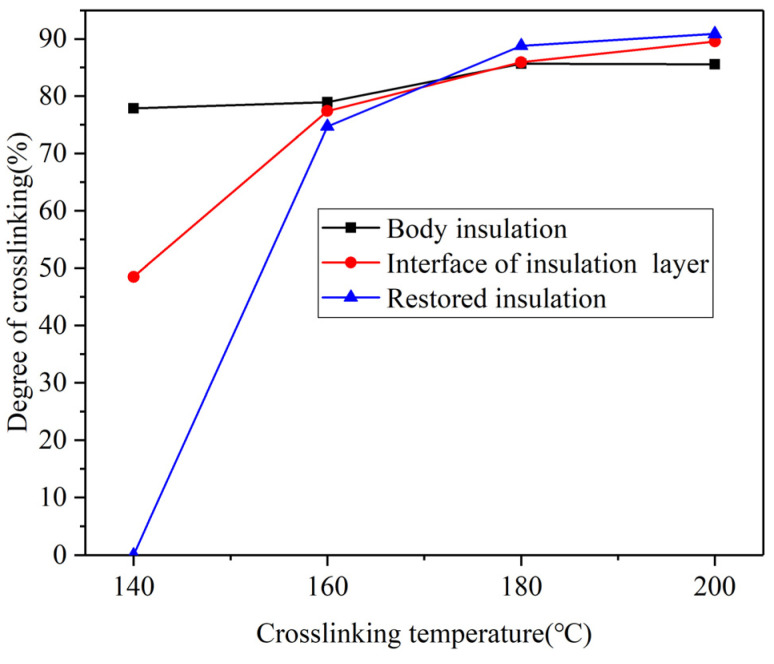
Crosslinking degree at different positions of the samples at various crosslinking temperatures.

The crosslinking degree of the XLPE sample was measured using the gel extraction method. The crosslinking degrees of the body insulation, insulation interface layer, and restored insulation at different crosslinking temperatures were calculated using Formula (1), as shown in Figure 5.

As shown in Figure 5, the crosslinking degree of both the insulation interface layer and the restored insulation increases with rising crosslinking temperature. In contrast, the crosslinking degree of the body insulation remains relatively constant with changes in crosslinking temperature. Specifically, the crosslinking degree of the insulation interface layer increases rapidly up to a crosslinking temperature of 180 °C, after which the increase slows down. The restored insulation is highly sensitive to crosslinking temperature. It exhibits a crosslinking degree of 0% at 140 °C, with a rapid increase in crosslinking degree as the temperature rises. The rate of increase in the crosslinking degree of the insulation interface layer falls between that of the body insulation and the recovery insulation.

### 3.2. Analysis of DSC Test Results

**Figure 6:**
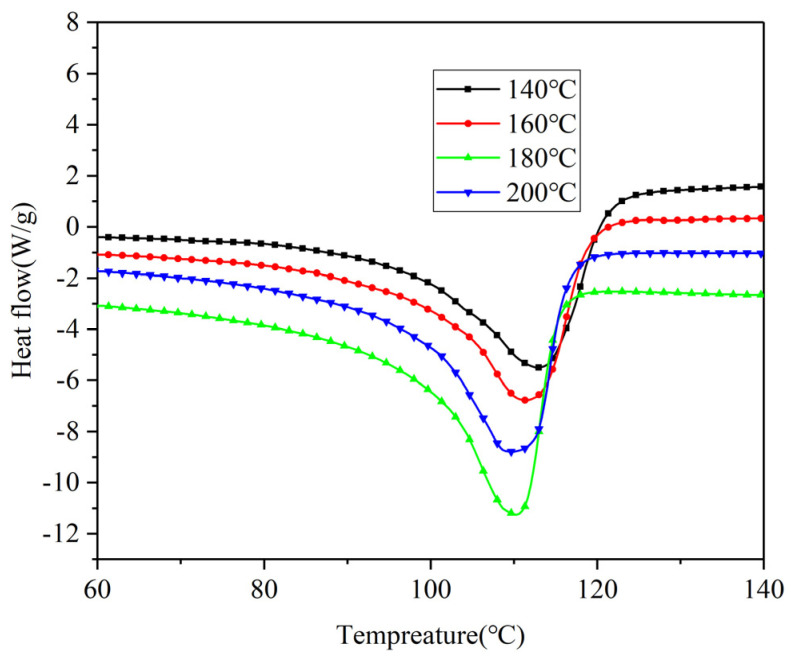
DSC curves of insulation interface samples at different crosslinking temperatures.

**Figure 7:**
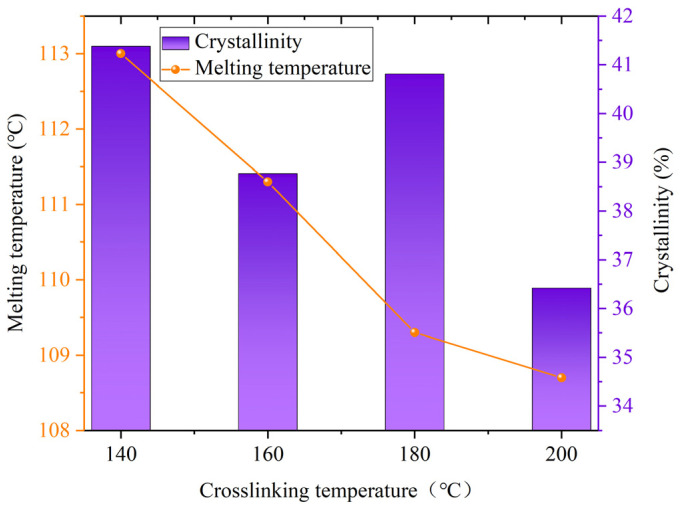
Melting temperature and crystallinity of insulation interface samples at different crosslinking temperatures.

To investigate the changes in crystallinity of XLPE insulation interface samples at different crosslinking temperatures, samples were prepared and tested following the procedure described in Section 2.3. The crystallinity of each group samples was then calculated. The DSC curves for each sample at various crosslinking temperatures are shown in Figure 6. To analyze the variations in crystallinity and melting temperature for each group of samples, calculations were performed based on the measured DSC curves. The specific results are presented in Figure 7.

As seen in Figure 6 and Figure 7, increasing the crosslinking temperature results in a decrease in the melting temperature of the insulation interface samples, accompanied by an overall decline in crystallinity. However, this trend is not monotonic. A peak in crystallinity occurs at a crosslinking temperature of 180 °C. This can be attributed to the limited decomposition of DCP at lower crosslinking temperatures, leading to low crosslinking efficiency. In this state, the uncrosslinked carbon chains exhibit better symmetry and enhanced crystal growth potential. As the crosslinking temperature rises, the decomposition rate of DCP accelerates, improving the crosslinking efficiency. Consequently, the original symmetry of the macromolecules is disrupted. The network structure formed by crosslinking reduces the regularity of molecular chains, inhibits crystal growth, and thereby decreases crystallinity. When the crosslinking temperature reaches 180 °C, a balance between the crosslinking degree and crystallinity is achieved, which enhances the crystalline morphology and results in a moderate increase in crystallinity. However, as the temperature continues to rise, excessive crosslinking temperature leads to an increased DCP decomposition rate, a higher crosslinking degree, and a reduced ability to form larger crystallinity domains, resulting in further decreases in crystallinity.

### 3.3. Analysis of Thermal Extension Experiment Results

**Figure 8:**
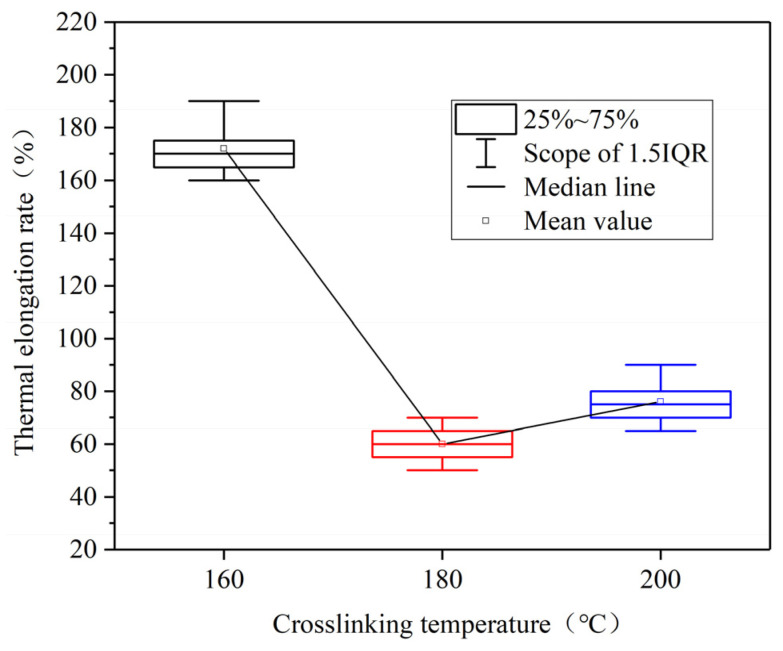
Thermal elongation at different crosslinking temperatures.

The thermal elongation of the insulation interface layer at different crosslinking temperatures was calculated using Formula (2), as shown in Figure 8. When the crosslinking temperature was 140 °C, all samples fractured during the thermal extension experiment. From Figure 8, it can be observed that the thermal elongation is minimal when the crosslinking temperature is 180 °C. Which is indicated that the sample’s performance is optimal at this temperature. However when the crosslinking temperature is increased to 200 °C, the thermal elongation increases, suggesting that a higher crosslinking temperature does not necessarily result in improved performance.

### 3.4. Analysis of Breakdown Voltage Test Results

**Figure 9:**
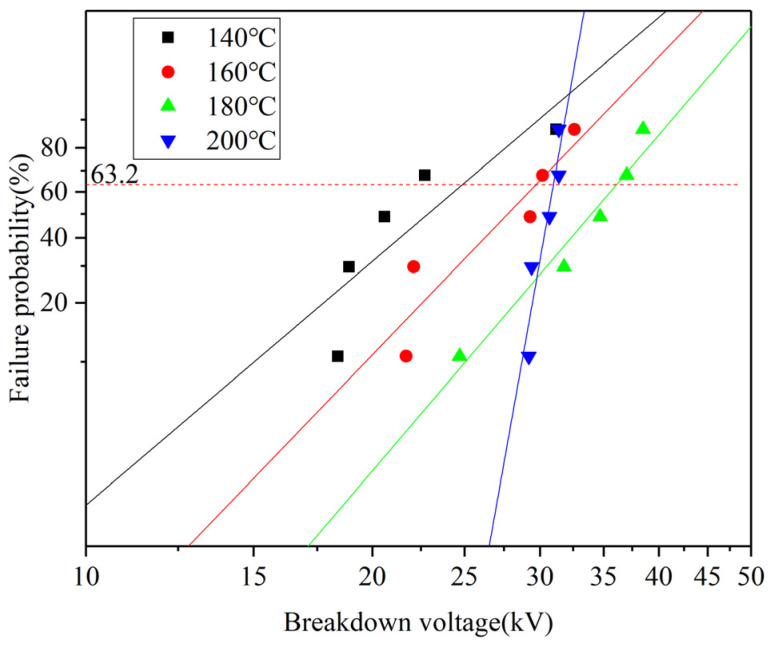
Relationship between breakdown voltage and failure probability at different crosslinking temperatures.

Power frequency breakdown experiments were conducted on samples at different crosslinking temperatures. The relationship between breakdown voltage and failure probability is presented in Figure 9. The breakdown voltage corresponding to a failure probability of 63.2% was taken as the characteristic breakdown voltage of each sample and is listed in Table 1. Comparing the characteristic breakdown voltage of interface samples at different crosslinking temperatures reveals a trend of initial increase followed by a decrease with rising crosslinking temperatures. When the crosslinking temperature is 180 °C, the characteristic breakdown voltage of interface samples is the highest, reaching to 36.23 kV. When the crosslinking temperature is 200 °C, the characteristic breakdown voltage of the interface sample decreases to 30.98 kV. When the crosslinking temperature is 140 °C, it is the lowest at 24.89 kV, significantly lower than the value at 180 °C. When the crosslinking temperature is 160 °C, the difference in characteristic breakdown voltage is relatively small compared to the 200 °C sample, but there is a notable increase in data dispersion.

### 3.5. Analysis of Scanning Electron Microscope Observation Results

**Figure 10:**
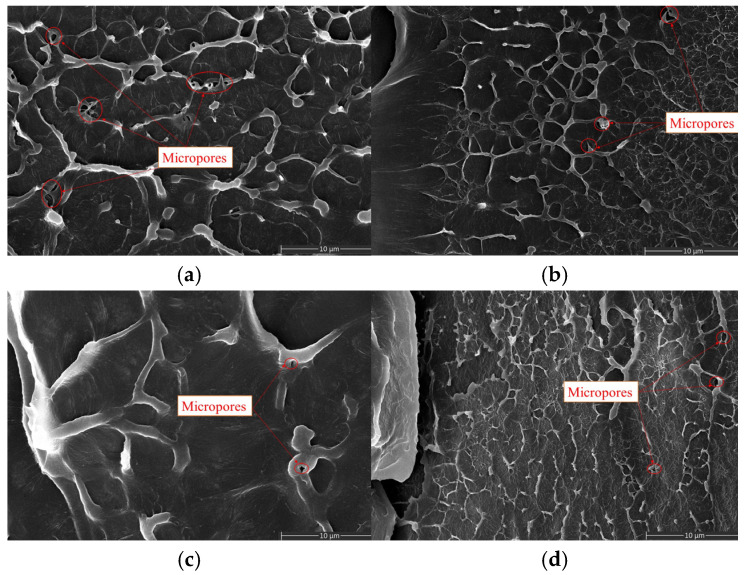
SEM images of insulation interface layer cross-sections at different crosslinking temperatures. (**a**) 140 °C (**b**) 160 °C (**c**) 180 °C (**d**) 200 °C.

After low-temperature quenching of insulation interface samples at different crosslinking temperatures, the quenched surface was observed using scanning electron microscopy at a magnification of 10,000 times. The results are shown in Figure 10a–d. The SEM images reveal the presence of micropores in the samples at all crosslinking temperatures. The maximum quantity and largest size of micropores were observed at a crosslinking temperature of 140 °C. As the crosslinking temperature increased, both the number and size of micropores decreased. When the crosslinking temperature was 180 °C, the number of micropores wasminimum. However, when the crosslinking temperature was 200 °C, although the size of the micropores was reduced, their number increased compared to the sample at 180 °C.

## 4. Discussion

**Figure 11:**
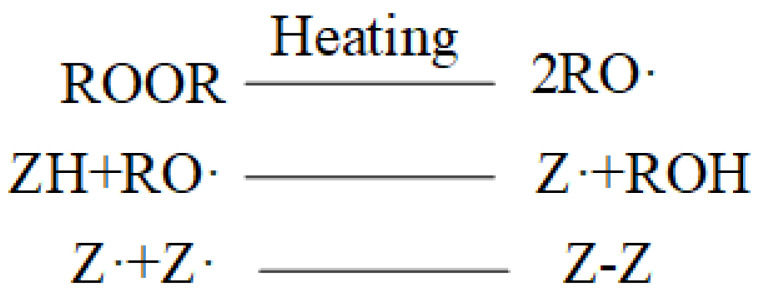
Schematic diagram of the crosslinking reaction [33].

XLPE is a high-performance polymer material modified by crosslinking polyethylene. The most commonly used crosslinking method in engineering involves the use of peroxides, which form a network structure of chemical bonds between polyethylene chains. The most widely used peroxide crosslinking agent is dicumyl peroxide (DCP), which decomposes upon heating during the crosslinking process. DCP decomposes upon heating to form free radicals, which take hydrogen atoms from the polyethylene chain and generate polyethylene macromolecular free radicals. Two polyethylene macromolecular free radicals then combine to form a cross-linked structure. The crosslinking reaction process is shown in Figure 11, where ROOR represents the peroxide crosslinking agent and ZH represents the PE long molecular chain.

**Figure 12:**
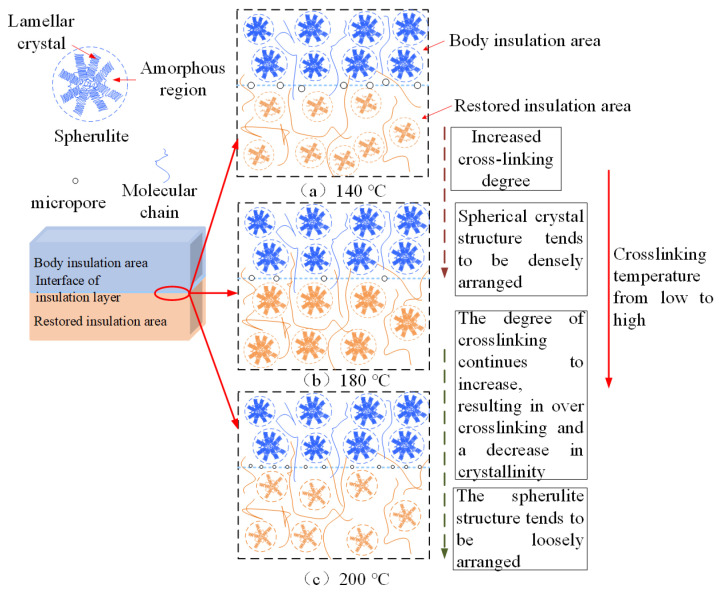
Aggregation structure model of insulation interface layer samples at different crosslinking temperatures.

This study primarily focuses on the insulation performance of the insulation interface layer, with particular attention to the changes in the crosslinking degree of this interface layer. According to the gel content test results, when the secondary crosslinking temperature is 140 °C, the gel content in the recovery insulation area is 0%. Although the crosslinking degree of the body insulation exceeds 80%, the crosslinking degree of the insulation interface layer at this temperature is approximately 40%. This suggests that DCP may not undergo sufficient thermal decomposition at this temperature, or the amount of decomposition is minimal. As a result, the un-crosslinking carbon chains exhibit good symmetry, facilitating strong crystal growth and resulting in a higher crystallinity of the sample. SEM observations further reveal that the insulation interface layer contains a significant number of micropores, with large pore diameters. Notably, all samples failed during the thermal extension test, and their characteristic breakdown voltage is found to be low. These findings are illustrated in Figure 12a.

As the secondary crosslinking temperature continues to rise until it reaches 180 °C, the DCP gradually decomposes, and the gel content of the insulation interface layer increases to approximately 85%, which is nearly the same as the gel content of the body insulation. At this time, the spherulite structure becomes more densely arranged, leading to an increase in the crystallinity. Simultaneously, the number of micropores in the insulation interface layer decreases, and their diameter becomes smaller. The thermal elongation gradually decreases to around 62%, while the characteristic breakdown voltage reaches the maximum value of 36.23 kV, as shown in Figure 12b. This phenomenon is similar to the conclusion that the insulation performance of XLPE insulation interface layer is the best when the crosslinking temperature is 180 °C [34].

When the secondary crosslinking temperature rises further to 200 °C, the gel content of the insulation interface layer increases to approximately 91%, surpassing that of the body insulation. This suggests the occurrence of over-crosslinking, which results in a decrease in crystallinity. SEM observations reveal that while the number of micropores increases, their diameters are smaller. As a result, the thermal elongation increases, but the characteristic breakdown voltage decreases. Nevertheless, the mechanical and electrical properties of the samples at 200 °C remain superior to those of samples crosslinked at temperatures below 160 °C, as illustrated in Figure 12c.

In summary, the increase of crosslinking temperature promotes an increase in the gel content of XLPE, causing the XLPE sample to transition from a linear molecular structure to a three-dimensional network structure. With the further rise in crosslinking temperature, the thermal elongation rate shows a trend of first decreasing and then increasing. The crystallinity shows a significant increasing trend at a crosslinking temperature of 180 °C. The characteristic breakdown voltage follows a trend of first increasing and then decreasing, which correlates with the observed microstructural changes.

## 5. Conclusions

This study investigates the impact of changes in secondary crosslinking temperature on the physical properties, electrical characteristics, and microstructure of XLPE insulation interface layer. The following conclusions have been drawn:

(1) Increasing the crosslinking temperature enhances the crosslinking degree of XLPE, which subsequently improves the crystal structure. When the secondary crosslinking temperature is 180 °C, the thermal elongation of the XLPE insulation interface sample is minimum and the characteristic breakdown voltage is maximum. An increase in the degree of crystallinity is observed. Therefore, increasing the crosslinking temperature can help improve the mechanical and electrical properties of XLPE insulation interface layers.

(2) When the crosslinking temperature reaches 200 °C, the thermal elongation increases, and the characteristic breakdown voltage decreases. Excessively high crosslinking temperatures lead to over-crosslinking, resulting in the formation of more amorphous regions and a decrease in crystallinity. It indicates that excessively high crosslinking temperature will reduce the insulation performance of the insulation interface layer.

## Figures and Tables

**Table 1:** Characteristic breakdown voltage of interface samples at different crosslinking temperatures.

Sample Type	Characteristic Breakdown Voltage/kV
140 °C crosslinking sample	24.89
160 °C crosslinking sample	29.72
180 °C crosslinking sample	36.23
200 °C crosslinking sample	30.98

## Data Availability

The original contributions presented in this study are included in the article. Further inquiries can be directed to the corresponding author.
